# Supplementary material for: The causal role of circulating inflammatory markers in osteoporosis: a bidirectional Mendelian randomized study
Source: Front Immunol. 2024 Jul 18;15:1412298. doi: 10.3389/fimmu.2024.1412298 (PMC11291241; doi:10.3389/fimmu.2024.1412298)
Supplement: Supplementary file 1 [file DataSheet_1.zip › supplementary material/CIM-OSTEOPOROSIS /GCST90274829.csv_leave_one_out.pdf]

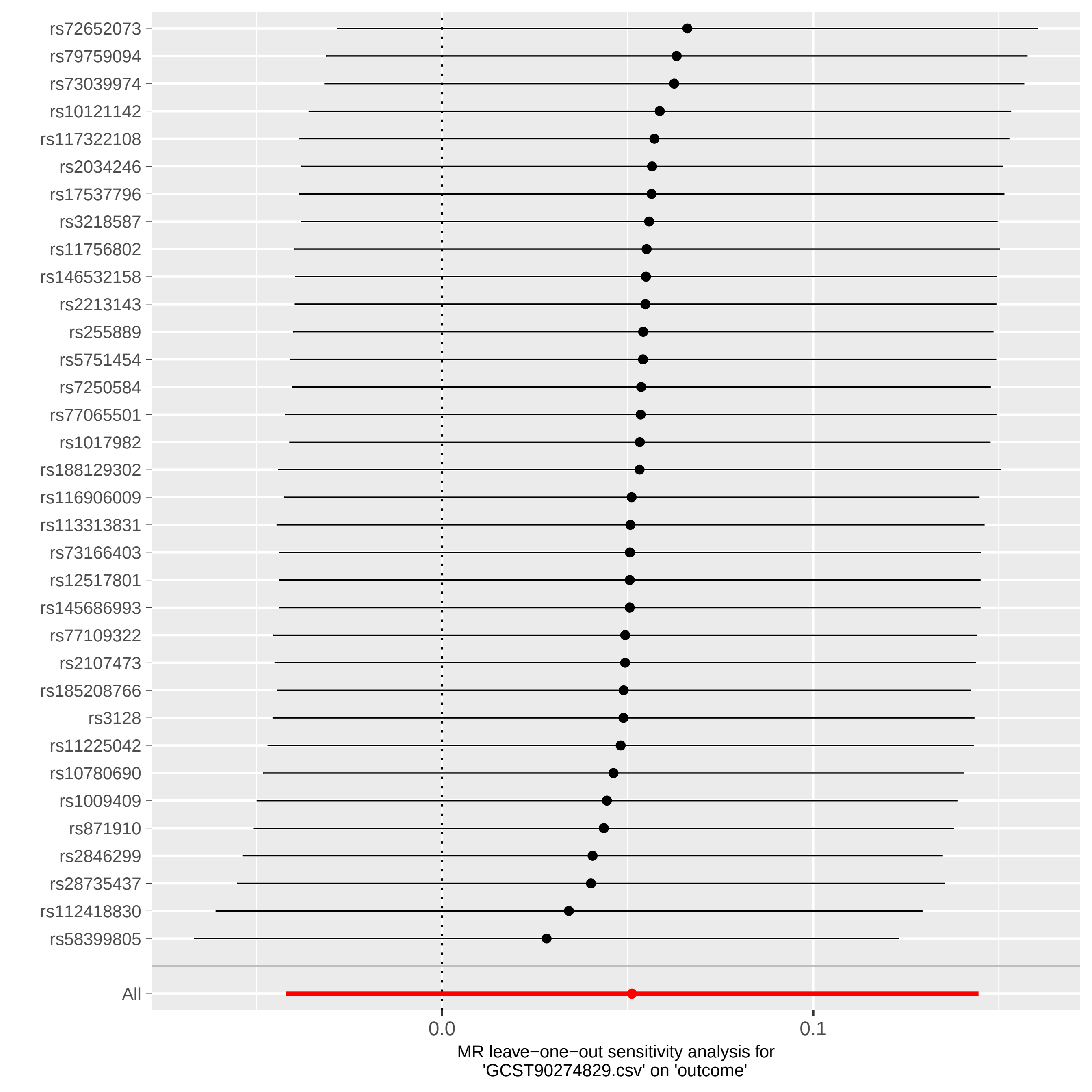

rs72652073
rs79759094
rs73039974
rs10121142
rs117322108
rs2034246
rs17537796
rs3218587
rs11756802
rs146532158
rs2213143
rs255889
rs5751454
rs7250584
rs77065501
rs1017982
rs188129302
rs116906009
rs113313831
rs73166403
rs12517801
rs145686993
rs77109322
rs2107473
rs185208766
rs3128
rs11225042
rs10780690
rs1009409
rs871910
rs2846299
rs28735437
rs112418830
rs58399805
All
0.0
0.1
MR leave−one−out sensitivity analysis for
'GCST90274829.csv' on 'outcome'
